# Spatiotemporal Patterns and Socioeconomic Determinants of Pulmonary Tuberculosis in Piauí, Northeast Brazil, 2001–2024

**DOI:** 10.1111/tmi.70133

**Published:** 2026-03-21

**Authors:** José Mário Nunes da Silva, Viriato Campelo, Walter Massa Ramalho

**Affiliations:** ^1^ Centre for Tropical Medicine, School of Medicine University of Brasília Brasília DF Brazil; ^2^ Laboratório de Inferência Causal Em Epidemiologia (LINCE‐USP), School of Public Health University of São Paulo São Paulo SP Brazil; ^3^ Núcleo de Ensino e Pesquisa em Tuberculose Federal University of Piauí Teresina PI Brazil; ^4^ Department of Parasitology and Microbiology Federal University of Piauí Teresina PI Brazil

**Keywords:** Bayesian analysis, Brazil, socioeconomic factors, spatial analysis, time series analysis, tuberculosis

## Abstract

**Background:**

Pulmonary tuberculosis (PTB) remains a major public health problem, strongly associated with social and territorial inequalities. This study aimed to analyse temporal trends, spatiotemporal transmission patterns and socioeconomic determinants of PTB in the state of Piauí, Northeast Brazil, from 2001 to 2024.

**Methods:**

An ecological study was conducted using official surveillance data on newly reported PTB cases at the municipal level. Temporal, spatial and spatiotemporal analytical methods were applied to investigate long‐term trends, transmission patterns and socioeconomic determinants associated with PTB incidence.

**Results:**

A total of 17,186 PTB cases were reported. Time‐series analysis showed a sustained decline in incidence between 2001 and 2015, followed by a period of gradual recovery starting in 2016. Seasonality remained stable, with peaks occurring between April and June. Persistent spatial heterogeneity was identified, with higher‐risk clusters concentrated in urban municipalities. Multivariate spatiotemporal time‐series analysis revealed the predominance of the endemic component, with a secondary contribution from the autoregressive component and minimal contribution from the spatiotemporal component. In the spatial Bayesian analysis, PTB incidence was associated with AIDS incidence (Relative Risk [RR] = 1.11; 95% Credible Interval [CrI]: 1.05–1.18), the number of nurses per 1000 inhabitants (RR = 1.08; 95% CrI: 1.03–1.13) and per capita municipal health expenditure (RR = 0.94; 95% CrI: 0.89–0.98).

**Conclusions:**

PTB in the Piauí exhibited heterogeneous spatiotemporal patterns, being sustained primarily by endemic factors and persistent structural inequalities. Control strategies should be territorially targeted, considering local transmission patterns and the identified determinants.

## 1 Introduction

Tuberculosis (TB) continues to pose a major global public health problem [1]. Although preventable and treatable, the disease continues to be strongly associated with social inequalities, poverty, and unequal access to health services, hindering the achievement of the elimination targets established by the World Health Organisation (WHO) [2]. In this context, Brazil is among the priority countries for TB control, with an incidence rate of 39.7 cases per 100,000 inhabitants, reflecting ongoing transmission and marked regional heterogeneity [3, 4].

Within Brazil, the state of Piauí, located in the Northeast region, presents an incidence rate below the national average; however, it continues to exhibit levels compatible with sustained transmission [3]. Recent evidence indicates an increasing trend in TB, particularly among younger age groups and Black and mixed‐race populations, with a predominance of the pulmonary form, the most transmissible, highlighting persistent social inequalities [5]. Furthermore, projections suggest that, if current conditions persist, the state is unlikely to achieve the TB reduction targets proposed by the WHO by 2035 [6].

Despite this scenario, up‐to‐date epidemiological studies examining pulmonary tuberculosis (PTB) in Piauí at the municipal level remain scarce. Most available investigations rely on analyses aggregated at the state level [4, 7, 8, 9], which limits the understanding of intrastate heterogeneity and hinders the planning of territorially targeted interventions that are sensitive to local inequalities.

In this regard, integrated analytical approaches are essential to capture the complex dynamics of PTB. The combination of temporal, spatial and spatiotemporal analyses allows the identification of trends, seasonality and high‐risk areas [4, 7], while explanatory spatial models help elucidate structural determinants associated with disease persistence [9, 10]. Such integration provides a comprehensive perspective on TB transmission and supports the development of more effective and equitable control strategies. Thus, this study aimed to analyse temporal trends, spatiotemporal transmission patterns, and socioeconomic determinants of PTB in the state of Piauí, Northeast Brazil, from 2001 to 2024.

## 2 Methods

### 2.1 Study Design

This is an ecological study using the 224 municipalities of the state of Piauí, Brazil, as the units of analysis, covering the period from 2001 to 2024.

### 2.2 Setting

**FIGURE 1:**
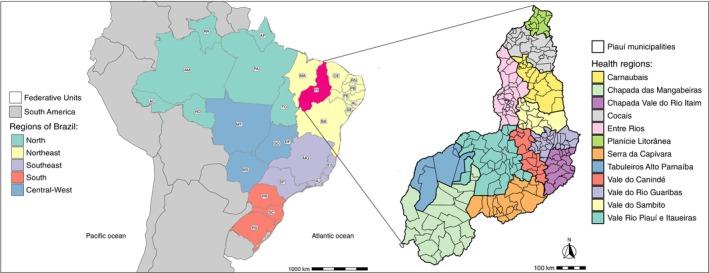
Geographic location of the study area, highlighting its 224 municipalities and 12 health regions, Piauí, Brazil.

Piauí, located in the Northeast region of Brazil, has approximately 3.27 million inhabitants distributed across 224 municipalities organised into 12 health regions (Figure 1). The state presents marked socioeconomic inequalities and has the second‐highest Gini coefficient in the country, estimated at 0.552, reflecting pronounced social and territorial contrasts for the analysis of PTB distribution [11].

### 2.3 Study Population and Data Source

The study included all newly reported PTB cases notified between 2001 and 2024 in the Brazilian Notifiable Diseases Information System (SINAN), which is managed and made publicly available by the Department of Informatics of the Unified Health System (DATASUS) (http://www2.datasus.gov.br).

We obtained population data from DATASUS, including census data from 2010 and 2022, as well as annual population estimates for the period from 2001 to 2024 produced by the Brazilian Institute of Geography and Statistics (IBGE) and integrated into the national database (https://www.gov.br/saude/pt‐br/composicao/seidigi/demas/dados‐populacionais).

### 2.4 Variables

The crude incidence rates were calculated by dividing the number of new cases by the resident population for each period and expressed the results per 100,000 inhabitants per year. Subsequently, we age‐standardised the incidence rates using the direct method, with 5‐year age groups, based on the standard population proposed by the WHO [12]. Cases with missing information on sex, age or municipality of residence were redistributed proportionally according to the distribution of available data [4].

We also considered demographic, socioeconomic, operational and epidemiological variables previously associated with municipal PTB incidence (Table S1) [13]. In addition, we included epidemiological characteristics of the reported cases.

### 2.5 Data Analysis

#### 2.5.1 Time Series Analysis

We conducted time series analysis using monthly PTB incidence rates, which we decomposed using the Seasonal and Trend decomposition using Loess (STL) method, implemented in R version 4.4.2 (R Foundation for Statistical Computing, Vienna, Austria). This approach allowed us to separate the series into trend, seasonal and residual components, providing a detailed characterisation of the temporal dynamics of PTB over the study period [14].

#### 2.5.2 Spatial and Spatiotemporal Analysis

We initially smoothed age‐standardised incidence rates using the local empirical Bayesian method to reduce instability associated with small populations and low case counts, particularly in smaller municipalities [15]. We then assessed global spatial autocorrelation using Moran's I, which quantifies spatial dependence by measuring similarity among neighbouring areas, ranging from −1 to +1 [16]. Statistical significance was evaluated using *z*‐scores and *p*‐values derived from 999 Monte Carlo permutations.

When significant spatial dependence was detected, we calculated Local Indicators of Spatial Association (LISA) to identify local clusters and spatial outliers. Municipalities were classified into high–high and low–low patterns, indicating positive spatial autocorrelation, as well as high–low and low–high patterns, reflecting spatial discordance and territorial disparities, based on their local spatial relationships [15]. All spatial analyses were conducted in the software R.

We performed space–time scan analysis with SaTScan version 10.3 (National Cancer Institute, Bethesda, MD, USA), based on a discrete Poisson model. Clusters were identified through moving cylindrical windows, in which the circular base represents the spatial dimension and the height corresponds to the temporal dimension [17].

The maximum spatial window was set to include up to 30% of the population at risk to avoid excessively large clusters and to ensure spatial specificity. The maximum temporal window was defined as 50% of the total study period. We also adjusted analyses for covariates, including age group and sex [18].

Clusters were ranked according to the log likelihood ratio, with the cluster presenting the highest value classified as the most likely cluster and remaining statistically significant, non‐overlapping clusters classified as secondary [18]. Statistical significance was assessed using 999 Monte Carlo simulations, and clusters with *p*‐values set at 0.05 were considered statistically significant.

#### 2.5.3 Spatiotemporal Analysis Using Multivariate Spatial Time Series Models

We performed spatiotemporal analysis of municipal surveillance data using multivariate spatial time series models in software R. We fitted the models to monthly case counts and specified a negative binomial regression (NegBin1) to account for overdispersion [19].

We defined the spatial structure of the epidemic component using a power‐law distance decay function [20]. The model decomposes the expected incidence into three components: an endemic component representing the baseline level of disease, an autoregressive component capturing within‐municipality temporal dependence, and a spatial epidemic component modelling transmission between municipalities over time. We also included seasonal effects and long‐term trends to capture periodic variation and gradual changes in incidence [19].

We estimated predicted values for each component along with their respective 95% confidence intervals (CIs). Further details are provided in the Supporting Information.

#### 2.5.4 Bayesian Spatial Analysis

We employed hierarchical Bayesian models to investigate demographic, socioeconomic, operational, and epidemiological factors associated with municipal PTB incidence. We initially fitted models without a spatial component and subsequently incorporated explicit spatial dependence using the Integrated Nested Laplace Approximation (INLA) approach [21].

We modelled municipal incidence assuming a Poisson distribution with a population offset, and we expressed effects as relative risks (RRs) with 95% credible intervals (CrI) (i.e., 2.5th and 97.5th percentiles) obtained from the posterior distributions. Prior to multivariable modelling, we assessed multicollinearity using the Spearman correlation matrix (Table S2) and the variance inflation factor (VIF), excluding highly collinear variables as shown in Table S3. We then standardised continuous covariates so that coefficients represented the effect associated with a one standard deviation (SD) increase.

We evaluated residual spatial autocorrelation in non‐spatial models using Moran's I, which supported the specification of spatial models. We incorporated spatial dependence using the BYM2 (Besag–York–Mollié) reparameterisation, which combines structured and unstructured spatial effects with penalised complexity priors for the proportion of variance attributed to the structured component and for global precision [22].

Multivariable spatial model selection was based on the Deviance Information Criterion (DIC) and complemented by the Watanabe Akaike Information Criterion (WAIC). A complete description of analytical procedures is provided in the Supporting Information. All analyses were conducted in R.

#### 2.5.5 Ethical Considerations

This study used exclusively publicly available, aggregated secondary data, with no access to individual‐level or identifiable information.

## 3 Results

Between 2001 and 2024, the state of Piauí reported 17,186 cases of PTB, of which 11,210 (65.2%) occurred in men, 10,518 (61.2%) among individuals of mixed race, and 7338 (42.7%) in the Entre Rios health region. The median age was 44 years (interquartile range [IQR]: 30–59). Detailed characteristics are presented in Table S4. The annual PTB incidence rate ranged from 42.6 per 100,000 inhabitants in 2001 to 19.6 per 100,000 in 2024, with an average rate of 24.8 per 100,000.

### 3.1 Time‐Series Analysis

**FIGURE 2:**
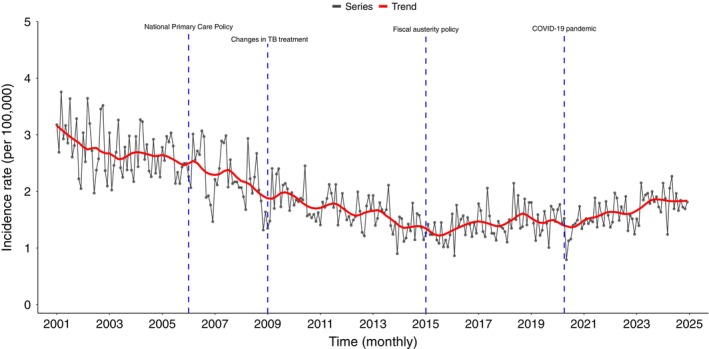
Monthly incidence of pulmonary tuberculosis in Piauí State, 2001–2024.

Analysis of monthly PTB incidence in Piauí revealed a long‐term trend characterised by a sustained decline between 2001 and 2015, followed by a period of gradual recovery from 2016 to 2024. Seasonality remained stable and recurrent throughout the entire series, with peaks concentrated between April and June, reaching a maximum in May and minimum values observed between December and February (Figure 2). A similar pattern was observed across health regions (Figure S1).

### 3.2 Spatial Patterns

**FIGURE 3:**
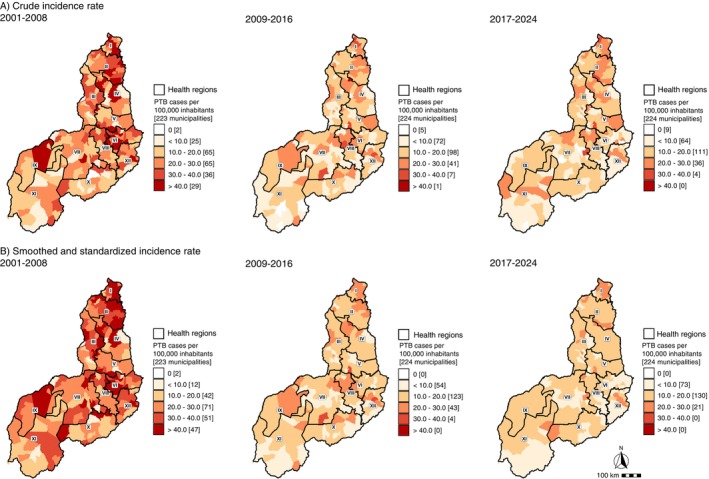
Spatial distribution of crude (A) and smoothed standardised (B) pulmonary tuberculosis incidence rates in the state of Piauí, Brazil, 2001–2024. Health regions: I–Planície Litorânea; II–Cocais; III–Entre Rios; IV–Carnaubais; V–Vale do Sambito; VI–Vale do Rio Guaribas; VII–Vale do Rio Piauí e Itaueiras; VIII–Vale do Canidé; IX–Tabuleiros do Alto Parnaíba; X–Serra da Capirava; XI–Chapada das Manguabeiras; and XII–Chapada Vale do Rio Itaim.

PTB in Piauí exhibited marked changes in spatial distribution across the analysed periods, considering crude and smoothed standardised rates (Figure 3). Global spatial autocorrelation analysis demonstrated significant spatial dependence throughout the entire period from 2001 to 2024. The global Moran's I was 0.356 (Z = 8.42; *p* = 0.001), with similar patterns observed across all analysed intervals, indicating persistent spatial clustering (Table S5).

**FIGURE 4:**
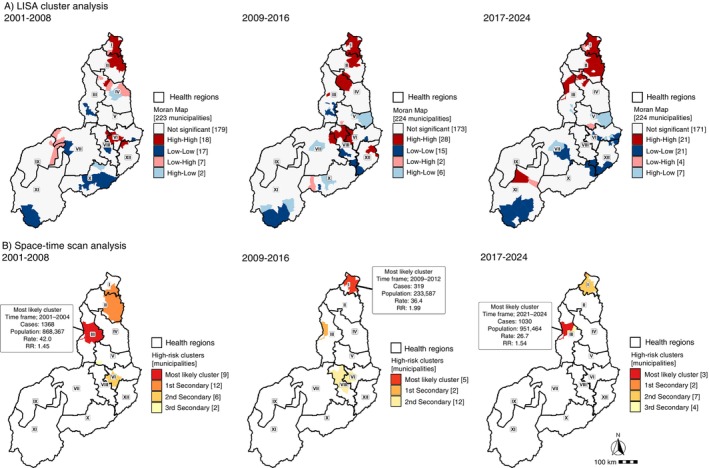
Local Indicators of Spatial Association (LISA) (A) and space–time scan analysis (B) of pulmonary tuberculosis in the state of Piauí, Brazil, 2001–2024. Health regions: I–Planície Litorânea; II–Cocais; III–Entre Rios; IV–Carnaubais; V–Vale do Sambito; VI–Vale do Rio Guaribas; VII–Vale do Rio Piauí e Itaueiras; VIII–Vale do Canidé; IX–Tabuleiros do Alto Parnaíba; X–Serra da Capirava; XI–Chapada das Manguabeiras; and XII–Chapada Vale do Rio Itaim.

Local spatial autocorrelation analysis identified 18 municipalities classified as high–high clusters during 2001–2008, primarily concentrated in the Vale do Rio Guaribas (*n* = 6), Planície Litorânea (*n* = 5), and Cocais (*n* = 4) health regions. Between 2009 and 2016, the number of high–high clusters increased by approximately 56%, totaling 28 municipalities, with the highest concentration in Planície Litorânea (*n* = 8), followed by Carnaubais (*n* = 6), Vale do Rio Guaribas (*n* = 5), and Cocais (*n* = 3). In the most recent period, 2017–2024, the number of high–high municipalities decreased by 25% relative to the previous period, totaling 21 clusters. These remained primarily concentrated in Planície Litorânea (*n* = 8), Cocais (*n* = 6), and Carnaubais (*n* = 4), while regions such as Vale do Rio Guaribas and Vale do Canindé no longer exhibited high–high clusters (Figure 4A).

### 3.3 Spatiotemporal Analysis

Space–time scan analysis identified four clusters during 2001–2008, three clusters during 2009–2016, and four clusters during 2017–2024 (Table 1). In the first period, the most likely cluster comprised nine municipalities, all located in the Entre Rios health region, including Teresina and municipalities within its metropolitan area. This cluster occurred between 2001 and 2004, comprising 1368 observed cases, with an incidence rate of 42.0 per 100,000 inhabitants and a RR of 1.45 (Figure 4B).

**TABLE 1:** Space–time scan analysis of pulmonary tuberculosis in the state of Piauí, Brazil, 2001–2024.

Cluster type	Time frame	Number of municipalities	Municipalities	Health region	Observed cases	Expected cases	Population	Rate^a^	RR	LLR	*p*‐Value
2001–2008											
Most likely	2001–2004	9	Alto Longá, Altos, Beneditinos, Coivaras, Demerval Lobão, Lagoa do Piauí, Monsenhor Gil, Pau D'arco do Piauí, Teresina	Entre Rios (9)	1368	1002.7	868,367	42.0	1.45	70.4	< 0.001
1st secondary	2003–2005	12	Brasileira, Domingos Mourão, Lagoa De São Francisco, Milton Brandão, Pedro II, Piracuruca, Piripiri, São João da Fronteira, São José do Divino | Caraúbas do Piauí, Cocal, Cocal dos Alves	Cocais (9), Planicie Litoranea (3)	300	175.9	196,766	52.5	1.74	37.2	< 0.001
2st secondary	2001–2004	6	Aroeiras do Itaim, Geminiano, Itainópolis, Paquetá, Picos, Santa Cruz do Piauí	Vale do Rio Guaribas (6)	219	122.4	98,405	55.0	1.81	31.5	< 0.001
3th secondary	2004–2004	2	Barra D'alcântara, Várzea Grande	Vale do Sambito (2)	16	2.7	8364	182.3	5.94	15.2	0.001
2009–2016											
Most likely	2009–2012	5	Bom Princípio do Piauí, Buriti Dos Lopes, Cocal, Luís Correia, Parnaíba	Planicie Litoranea (5)	319	165.9	233,587	36.4	1.99	58.0	< 0.001
1st secondary	2009–2012	2	Demerval Lobão, Teresina	Entre Rios (2)	867	608.0	855,657	27.0	1.52	56.7	< 0.001
2st secondary	2009–2012	12	Campinas do Piauí, Colônia do Piauí, Floresta do Piauí, Oeiras, Santo Inácio do Piauí, São João da Varjota | Aroeiras do Itaim, Dom Expedito Lopes, Paquetá, Picos, Santa Cruz do Piauí, Wall Ferraz	Vale do Caninde (6), Vale do Rio Guaribas (6)	180	119.9	161,722	28.4	1.52	13.4	0.005
2017–2024											
Most likely	2021–2024	3	Altos, Demerval Lobão, Teresina	Entre Rios (3)	1030	721.7	951,464	27.6	1.54	69.5	< 0.001
1st secondary	2021–2024	2	Ilha Grande, Parnaíba	Planicie Litoranea (2)	238	132.8	174,009	33.5	1.83	34.8	< 0.001
2st secondary	2017–2019	7	Bom Princípio do Piauí, Buriti dos Lopes, Caraúbas Do Piauí, Caxingó, Cocal, Murici dos Portelas, Parnaíba	Planicie Litoranea (7)	205	126.8	240,174	30.2	1.64	20.9	< 0.001
3th secondary	2017–2019	4	Altos, Coivaras, Pau D'arco do Piauí, Teresina	Entre Rios (4)	639	502.6	943,503	23.8	1.31	19.1	< 0.001

Abbreviations: LLR, log‐likelihood ratios; RR, relative risk.

^a^ Age‐ and sex‐standardised incidence rate per 100,000 inhabitants‐years.

During 2009–2016, the most likely cluster shifted to the Planície Litorânea, encompassing five municipalities between 2009 and 2012. This cluster comprised 319 observed cases, with an incidence rate of 36.4 per 100,000 and an RR of 1.99 (Figure 4B). In the period 2017–2024, the most likely cluster returned to the Entre Rios health region, involving Altos, Demerval Lobão, and Teresina between 2021 and 2024. This cluster comprised 1030 observed cases, with an incidence rate of 27.6 per 100,000 and an RR of 1.54 (Figure 4B). Detailed information on all secondary clusters is presented in Table 1.

### 3.4 Spatiotemporal Analysis of Multivariate Spatial Time Series Model

A multivariate spatial time‐series model was fitted to monthly PTB data from 2001 to 2024 (Table S6). Observed incidence was decomposed into three epidemiologically interpretable components: endemic, autoregressive, and spatiotemporal transmission between neighbouring municipalities. Mean monthly total effects indicated that the endemic component accounted for the majority of the disease burden, averaging 49.9 (95% CI: 48.8–51.1) cases per month. The autoregressive component contributed a smaller yet non‐negligible share, averaging 4.9 (95% CI: 4.3–5.7) cases per month, while the spatiotemporal component between municipalities remained negligible throughout the study period. The model also identified a statistically significant decreasing temporal trend, corresponding to an average monthly reduction of 0.27% (95% CI: 0.25%–0.30%) after adjustment for seasonality and other components.

Comparison of fitted components across municipalities revealed marked spatial heterogeneity and consistency with the spatiotemporal scan results. Decomposition plots for the 15 municipalities with the highest mean contributions are presented in Figure S2.

### 3.5 Factors Associated With Pulmonary Tuberculosis

The non‐spatial multivariable Bayesian model identified statistically significant associations between PTB incidence and socioeconomic and operational variables, including unemployment rate, adult illiteracy, number of nurses per 1000 inhabitants, and AIDS incidence (Table S6). However, model diagnostics indicated substantial overdispersion (ĉ = 2.67; 95% CrI: 2.36–3.04) and significant spatial autocorrelation in the residuals (Moran's I = 0.26; *p* = 0.001) (Table S7).

Incorporation of the spatial component resulted in improved global model fit, with reduced information criteria (DIC = 1550.99; WAIC = 1540.90) compared with the non‐spatial model (DIC = 1800.23; WAIC = 2232.34). In the multivariable spatial model, PTB incidence remained positively associated with the number of nurses per 1000 inhabitants (RR = 1.08; 95% CrI: 1.03–1.13) and AIDS incidence (RR = 1.11; 95% CrI: 1.05–1.18), while municipal per capita health expenditure showed an inverse association (RR = 0.94; 95% CrI: 0.89–0.98) (Table 2).

**TABLE 2:** Effect estimates from univariate and multivariable Bayesian spatial models of pulmonary tuberculosis determinants in the state of Piauí, Brazil, 2001–2024.

Variables	Mean (SD)	RR	95% CrI	DIC	WAIC
Univariate models for PTB					
Demographic					
Sex ratio (M/F)	101.3 (4.8)	0.87	0.82–0.92	1555.41	1544.87
Proportion of individuals aged 65+	9.3 (11.7)	1.02	0.96–1.09	1559.79	1543.69
Proportion of Black and Brown population	0.79 (0.09)	0.99	0.92–1.06	1560.17	1544.42
Household crowding	32.3 (6.7)	0.93	0.87–0.99	1558.84	1544.25
Adequate sanitation coverage (%)	15.1 (15.8)	1.00	0.95–1.06	1560.45	1544.79
Socioeconomic					
Municipal Human Development Index	0.57 (0.04)	1.10	1.04–1.15	1558.49	1546.57
Gini index (%)	54.0 (0.05)	0.97	0.92–1.03	1560.64	1545.05
Social Vulnerability Index	0.47 (0.07)	0.90	0.86–0.95	1558.94	1547.17
Illiteracy rate of those aged ≥ 18 year	32.9 (6.6)	0.93	0.87–0.98	1561.57	1549.30
Unemployment rate	7.7 (4.7)	1.02	0.96–1.08	1560.52	1544.66
% of vulnerable to poverty	69.5 (8.3)	0.89	0.84–0.93	1555.51	1544.93
Average household income per capita (US$)	229.2 (8.8)	1.11	1.06–1.16	1554.35	1541.59
GDP per capita (US$)	3019.1 (3564.2)	1.09	1.03–1.15	1559.79	1546.24
Families benefiting from *Bolsa Família*	2696.2 (7233.8)	1.05	1.01–1.09	1558.56	1543.43
Operational and epidemiological					
Number of nurses (per 1000 inhabitants)	1.4 (0.64)	1.12	1.07–1.17	1558.15	1548.04
Number of physicians (per 1000 inhabitants)	0.9 (0.6)	1.10	1.06–1.15	1556.59	1544.87
Coverage of primary care (%)	99.6 (1.48)	1.03	0.97–1.08	1559.38	1543.18
AIDS incidence rate (per 100,000 inhabitants)	5.1 (4.2)	1.17	1.06–1.15	1555.58	1544.68
Municipal health expenditure (per capita)	248.4 (71.0)	0.97	0.91–1.01	1558.67	1542.32
Multivariable model for PTB					
Operational and epidemiological				1550.99	1540.90
Number of nurses (per 1000 inhabitants)	1.4 (0.64)	1.08	1.03–1.13		
AIDS incidence rate (per 100,000 inhabitants)	5.1 (4.2)	1.11	1.05–1.18		
Municipal health expenditure (per capita)	248.4 (71.0)	0.94	0.89–0.98		
Random effects, median (95% CrI)					
Global precision (τ)	8.45	5.93–12.01			
Spatial fraction (φ)	0.60	0.29–0.85			
Spatially unstructured residual (τᵥ)	21.5	13.1–48.0			
Spatially structured residual (τᵤ)	14.1	7.5–36.7			
Overdispersion (ĉ)	1.21	0.89–1.72			
Global Moran's I, *p*‐value	−0.05	0.880			

*Note:* Effect estimates represent the relative change in the incidence rate associated with a one–standard deviation (1 SD) increase in the exposures of interest. RRs with 95% CrI excluding 1 were considered indicative of a statistically significant.

Abbreviations: CrI: credible interval; DIC: deviance information criterion; PTB: pulmonary tuberculosis; RR: relative risk; SD: standard deviation; WAIC: Watanabe–Akaike information criterion.

Decomposition of the spatial random effects indicated substantial residual spatial heterogeneity, with about 60% of the variance explained by the structured spatial component (Table 2). The inclusion of spatial effects markedly reduced overdispersion and eliminated residual spatial autocorrelation, indicating good model fit (Figure S4) and adequate capture of spatial dependence in PTB incidence (Table 2).

## 4 Discussion

This study provides an integrated assessment of the temporal, spatial, and spatiotemporal patterns of PTB in the state of Piauí between 2001 and 2024, as well as its associated determinants. A sustained decline in monthly PTB incidence was observed from 2001 to 2015, followed by a gradual recovery from 2016 onward and increased instability in more recent years, indicating an inflection in the historical downward trend of the disease in the state, and corroborating findings from recent studies conducted in the region [4, 5].

This shift can be interpreted in the context of broader structural changes in Brazil's political, economic, and health‐care landscape. The period after 2015 coincides with the intensification of fiscal austerity policies and budgetary constraints that affected the financing and operational capacity of the Unified Health System (SUS), particularly in more vulnerable states [23]. Reduced investments in primary health care, epidemiological surveillance, and active case‐finding may have weakened TB control strategies [24]. This scenario was further aggravated from 2020 onward by the COVID‐19 pandemic, which disrupted routine health services, reduced case detection, and caused diagnostic delays, followed by a delayed recovery of services. Together, these factors likely contributed to the increased variability and partial reversal of the downward PTB trend observed in recent years [25].

At the same time, part of the post‐2016 increase in incidence may reflect improvements in diagnostic capacity rather than a true resurgence of transmission. The progressive expansion of bacteriological and molecular diagnostic tools, particularly sputum microscopy and Xpert MTB/RIF, implemented in Piauí after 2014, may have enhanced case detection and reduced underdiagnosis [5, 26], influencing the observed temporal dynamics.

The seasonal pattern identified in this study suggests that PTB incidence in Piauí varies throughout the year and is strongly influenced by the organisation and functioning of health services [27]. Peaks in case detection may be related to intensified active case‐finding and awareness campaigns [28], such as those surrounding World TB Day, as well as increased health‐care utilisation during intermediate months of the year, when respiratory infections are more frequent [29]. Conversely, the decline observed at the end of the year likely reflects temporary reductions in service availability and utilisation rather than a true decrease in transmission [4], underscoring the importance of maintaining continuous TB control efforts year‐round.

The multivariate spatial time‐series analysis revealed the predominance of the endemic component across all municipalities in Piauí, indicating that the PTB burden is mainly sustained by persistent structural factors [10]. The autoregressive component contributed secondarily, while the spatiotemporal component related to transmission between municipalities was minimal. This pattern suggests that PTB dynamics in the state are less driven by short‐term outbreaks or recent spatial diffusion and are instead shaped by long‐standing social, economic, and health‐system conditions.

Similarly, patterns have been reported in studies from China [10], where multivariate spatial time‐series models also identified the endemic component as the main driver of TB incidence, with smaller contributions from autoregressive effects and limited influence of spatial transmission between administrative units. These convergent findings reinforce the interpretation of TB as a chronic endemic condition anchored in structural determinants rather than driven by episodic spatial spread.

In the context of Piauí, these results are consistent with previous temporal decomposition analyses showing that, after notable epidemiological gains up to the mid‐2010s, structural factors regained prominence in shaping disease incidence in subsequent years [5]. By explicitly incorporating the spatial dimension, the present study advances understanding of PTB persistence in the state, highlighting the central role of territorial inequalities, socioeconomic vulnerabilities, and structural limitations in health service organisation.

This interpretation is further supported by the space–time scan analysis. Municipalities with higher mean endemic component values, such as Teresina, Parnaíba, Picos and Piripiri, were consistently identified among the most likely and secondary clusters across all three study periods. Teresina, in particular, was included in almost all likely clusters during 2001–2008 and 2017–2024, concentrating a large number of cases and maintaining relative risks persistently above the state average. Parnaíba showed a similar pattern from 2009 onward, while Picos and Piripiri emerged as relevant secondary clusters in the interior. The strong concordance between endemic component dominance and persistent spatiotemporal clusters indicates coherence between the different analytical approaches used.

From a territorial perspective, these clusters were predominantly located in municipalities with higher population density, greater urbanisation, and elevated social vulnerability, particularly in the capital and coastal areas [8, 13, 30]. These findings reinforce that PTB incidence in the state is not randomly distributed but reflects the interaction of structural factors that sustain persistent spatial patterns of higher risk.

The determinants identified in the multivariate spatial model help explain why municipalities with higher endemic contributions also emerged as persistent high‐risk clusters. The positive association between PTB incidence and the number of nurses per 1000 inhabitants may reflect greater surveillance sensitivity and case‐detection capacity in municipalities with more structured health systems [31]. However, in ecological studies, workforce organisation may also respond to the pre‐existing disease burden, limiting causal interpretation and suggesting the possibility of reverse causality [10]. Because diagnostic effort could not be directly measured by municipality of residence, this association should be interpreted with caution, as it may reflect both surveillance effects and residual structural and epidemiological factors.

In contrast, the inverse association with municipal per capita health expenditure suggests that higher levels of public investment contribute to sustained reductions in PTB incidence by strengthening preventive actions, expanding timely access to diagnosis and ensuring treatment continuity, particularly in socially vulnerable populations [32]. Evidence from recent studies indicates that investments in TB control are cost‐effective and yield substantial reductions in cases and deaths, as well as high social returns per dollar invested [33, 34].

Finally, the consistent association with AIDS incidence underscores the ongoing importance of TB–HIV coinfection in sustaining the disease burden, especially in urban and socially vulnerable settings [9, 35]. Taken together, these findings indicate that persistent spatial clusters of PTB in Piauí arise from the interaction of socioeconomic inequalities, epidemiological vulnerability, and health system capacity, suggesting that disease persistence is driven predominantly by chronic structural conditions rather than short‐lived spatial events.

## 5 Limitations and Strengths

This study has some limitations. It relies on secondary surveillance data, which are subject to underreporting, diagnostic delays and recording inconsistencies. The lack of socioeconomic and operational covariates with continuous temporal variation over the entire study period precluded spatiotemporal modelling with time‐varying determinants, restricting the analysis of associated factors to a predominantly spatial framework. In addition, the use of municipality‐level aggregated indicators may mask important intraurban inequalities, especially in larger municipalities, and the findings are subject to ecological fallacy, precluding causal inference at the individual level.

Another limitation is that several socioeconomic determinants were derived from the 2010 Brazilian Census, while the epidemiological analysis extends to 2024. Although the 2022 census has been conducted, detailed municipal‐level socioeconomic indicators are not yet fully available. While these variables capture long‐term structural inequalities that tend to change gradually, socioeconomic conditions may have evolved over the past decade. However, the relative socioeconomic differences between municipalities are likely to have remained proportionally stable, allowing these indicators to serve as reasonable proxies for structural inequalities across the study period.

Key strengths include an integrated analytical approach combining time‐series analysis using STL and multivariate spatial time‐series, spatial and spatiotemporal methods, and Bayesian spatial modelling, providing a comprehensive assessment of PTB dynamics over more than two decades. The use of monthly municipal‐level data enabled the identification of fine‐scale temporal patterns, persistent territorial heterogeneity, and spatiotemporal clusters of risk, surpassing analyses restricted to the state level. Although conducted in Piauí, both the findings and the methodological framework are potentially generalisable to other settings with similar socioeconomic and epidemiological characteristics, supporting evidence‐based planning in regions marked by social and territorial inequalities.

## 6 Conclusion

The findings indicate that, despite relevant advances, PTB transmission in Piauí remains shaped by pronounced territorial inequalities and high sensitivity to broader social, economic and institutional changes. The integration of long‐term temporal analysis, identification of persistent spatial clusters, and assessment of structural determinants provides a robust analytical framework to guide TB control strategies that are both geographically targeted and socially informed. Strengthening primary health care, reducing socioeconomic inequalities, and enhancing the capacity and resilience of epidemiological surveillance systems are essential to interrupt persistent transmission and achieve sustained progress toward TB elimination goals.

## Funding

The authors have nothing to report.

## Conflicts of Interest

The authors declare no conflicts of interest.

## Supporting information


**Table S1.** Description of the socioeconomic indicators used in the study.
**Table S2.** Spearman correlation matrix of the social determinants analysed in the study.
**Table S3.** Variance inflation factors and tolerance before and after exclusion of collinear variables.
**Table S4.** Epidemiological characteristics of pulmonary tuberculosis cases in the state of Piauí, Brazil, 2001–2024.
**Table S5.** Global spatial autocorrelation analysis of pulmonary tuberculosis in the state of Piauí, Brazil, 2001–2024.
**Figure S1.** Monthly incidence of pulmonary tuberculosis by health regions in Piauí State, 2001–2024.
**Figure S2.** Fitted components in the multivariate time series model for the 15 municipalities with the highest mean contributions in Piauí State, 2001–2024.
**Table S6.** Estimated components of the multivariate time‐series model for pulmonary tuberculosis incidence in Piauí, Brazil, 2001–2024.
**Figure S3.** Probability integral transform (PIT) histogram for time‐series model calibration.
**Table S7.** Effect estimates from univariate and multivariable Bayesian non‐spatial models of pulmonary tuberculosis determinants in the state of Piauí, Brazil, 2001–2024.
**Figure S4.** Observed versus fitted values from the Bayesian spatial model for pulmonary tuberculosis incidence in Piauí, Brazil, 2001–2024.

## Data Availability

The data that support the findings of this study are openly available in Department of Informatics of the Unified Health System at https://datasus.saude.gov.br/.
