# Supplementary material for: Spatiotemporal Patterns and Socioeconomic Determinants of Pulmonary Tuberculosis in Piauí, Northeast Brazil, 2001–2024
Source: Trop Med Int Health. 2026 Mar 21;31(6):754–65. doi: 10.1111/tmi.70133 (PMC13246586; doi:10.1111/tmi.70133)
Supplement: Supplementary file 1 — Table S1. Description of the socioeconomic indicators used in the study. Table S2. Spearman correlation matrix of the social determinants analysed in the study. Table S3. Variance inflation factors and tolerance before and after exclusion of collinear variables. Table S4. Epidemiological characteristics of pulmonary tuberculosis cases in the state of Piauí, Brazil, 2001–2024. Table S5. Global spatial autocorrelation analysis of pulmonary tuberculosis in the state of Piauí, Brazil, 2001–2024. Figure S1. Monthly incidence of pulmonary tuberculosis by health regions in Piauí State, 2001–2024. Figure S2. Fitted components in the multivariate time series model for the 15 municipalities with the highest mean contributions in Piauí State, 2001–2024. Table S6. Estimated components of the multivariate time‐series model for pulmonary tuberculosis incidence in Piauí, Brazil, 2001–2024. Figure S3. Probability integral transform (PIT) histogram for time‐series model calibration. Table S7. Effect estimates from univariate and multivariable Bayesian non‐spatial models of pulmonary tuberculosis determinants in the state of Piauí, Brazil, 2001–2024. Figure S4. Observed versus fitted values from the Bayesian spatial model for pulmonary tuberculosis incidence in Piauí, Brazil, 2001–2024. [file TMI-31-754-s001.docx]

**Supplemental Material**

**Spatiotemporal patterns and socioeconomic determinants of pulmonary tuberculosis in Piauí, Northeast Brazil, 2001–2024**

*Tropical Medicine & International Health*

**Table of contents**

[**Supplementary methods 2**](#_Toc218808230)

[**Spatiotemporal analysis using multivariate spatial time-series models 5**](#_Toc218808231)

[**Endemic component and seasonality 5**](#_Toc218808232)

[**Epidemic components 6**](#_Toc218808233)

[**Spatial connectivity 6**](#_Toc218808234)

[**Bayesian spatial analysis of the determinants of pulmonary tuberculosis 7**](#_Toc218808235)

[**Non-spatial Bayesian models 7**](#_Toc218808236)

[**Spatial Bayesian models 8**](#_Toc218808237)

[**Supplementary results 10**](#_Toc218808238)

[**References 23**](#_Toc218808239)

# Supplementary methods

**Table S1.** Description of the socioeconomic indicators used in the study.

| **Category** | **I****ndicators** | **Description** | **Year** | **Data source** |
| --- | --- | --- | --- | --- |
| Demographic | Sex ratio | Ratio of the male population to the female population in the municipality. | 2022 | IBGE [1] |
|  | Proportion of individuals aged 65+ | Percentage of the municipal population aged 65 years or older. | 2022 | IBGE [1] |
|  | Proportion of Black and Brown population | Percentage of residents self-identifying as Black or Brown (pardo), according to census classification. | 2022 | IBGE [1] |
|  | Household crowding | Ratio of the population living in private households with a density greater than 2 to the total population residing in private households, multiplied by 100. The household density is given by the ratio of the total number of residents to the total number of rooms used as bedrooms. | 2010 | HDA [2] |
|  | Adequate sanitation coverage (%) | Percentage of households with access to adequate sanitation services, including sewerage and safe waste disposal. | 2010 | IBGE [3] |
| Socioeconomic | Municipal Human Development Index | Geometric mean of the indices for the dimensions of Income, Education, and Longevity, with equal weights. The scale ranges from 0 (worst) to 1 (best). | 2010 | HDA [2] |
|  | Gini Index (%) | It measures the degree of income inequality within a given group, indicating the disparity between the incomes of the poorest and the richest. It is calculated as a ratio of the areas in the Lorenz curve diagram. | 2010 | HDA [2] |
|  | Social Vulnerability Index | Arithmetic means of the indices for the dimensions: urban infrastructure index, human capital index, and income and employment index. The scale ranges from 0 (best) to 1 (worst). | 2010 | HDA [2] |
|  | Illiteracy rate of those 18 years and older | Ratio of the population aged 18 or older who cannot read or write a simple note to the total number of people in that age group, multiplied by 100. | 2010 | HDA [2] |
|  | Unemployment rate | Percentage of the economically active population (EAP) in that age group who were unemployed, i.e., those who were not employed in the week prior to the census date but had been seeking work in the month leading up to the survey. | 2010 | HDA [2] |
|  | % of vulnerable to poverty | Percentage of people with per capita household income equal to or below R$255.00 per month (US$145), in August 2010 reais, equivalent to half the minimum wage. | 2010 | HDA [2] |
|  | GDP per capita (US$) | Sum of municipal assets divided by the total municipal population. Values are in Brazilian reais (R$5.14), converted to US dollars ($1.00) as of August 1, 2021. | 2021 | IBGE [4] |
|  | Average household income per capita (US$) | Ratio of the total income of all individuals residing in permanent private households to the total number of these individuals. Values are in Brazilian reais (R$1.80), converted to US dollars ($1.00) as of August 1, 2010. | 2010 | HDA [2] |
|  | Families benefiting from Bolsa Família | Number of households receiving benefits from the Brazilian conditional cash transfer program Bolsa Família. | 2023 | MSD [5] |
| Operational and epidemiological | Number of nurses | Density of registered nurses per 1,000 residents in the municipality. | 2023 | CNES [6] |
|  | Number of physicians | Density of registered physicians per 1,000 residents in the municipality. | 2023 | CNES [6] |
|  | Primary health care coverage (%) | Estimate of the population covered by primary care teams, multiplied by 100. | 2023 | e-Gestor [7] |
|  | AIDS incidence rate | Number of new AIDS cases in a specific population of a municipality during a defined period of time (cases per 100,000 inhabitants). | 2001-2024 | SINAN [8] |
|  | Municipal health expenditure (per capita) | Annual public health expenditure per resident at the municipal level. | 2023 | SIOPS [9] |

*Abbreviations:* CNES, National Registry of Health Facilities (Cadastro Nacional dos Estabelecimentos de Saúde, in Portuguese); GDP, Gross Domestic Product; HDA, Human Development Atlas in Brazil (Atlas do Desenvolvimento Humano do Brasil, in Portuguese); IBGE, Brazilian Institute of Geography and Statistics (Instituto Brasileiro de Geografia e Estatística, in Portuguese); e-Gestor, Primary Health Care Information System (Sistema de Informação e Gestão da Atenção Básica, in Portuguese); MSD, Ministry of Social Development and Fight Against Hunger; SINAN, Notifiable Diseases Information System (Sistema de Informação de Agravos de Notificação, in Portuguese); SIOPS, Public Health Budget Information System (Sistema de Informações sobre Orçamentos Públicos em Saúde, in Portuguese).

## **Spatiotemporal analysis using multivariate spatial time-series models**

To investigate the spatiotemporal dynamics of pulmonary tuberculosis across municipalities in the state of Piauí, Brazil, we fitted a multivariate time-series model for count data using the ***hhh4* framework** implemented in the surveillance package in R (version 4.4.2. R Foundation for Statistical Computing, Vienna, Austria) [10]. Let *Y_i_*​ denote the number of reported tuberculosis cases in municipality *i* = 1,…,*I*, at time *t* = 1,…,*T*. Conditional on the past information $F_{t-1}$​, the counts were assumed to follow a **negative binomial distribution of type NegBin1** [11]:

$$Y_{i,t}\mid\mathcal{F}_{t-1}\sim\text{NegBin}\left( \mu_{i,t},\psi\right),$$

where $\mu_{i,t}$ is the conditional mean and *ψ* is the overdispersion parameter. Under the NegBin1 parameterization, the conditional variance is given by:

$$\text{Var}\left( Y_{i,t} \right)=\mu_{i,t}\left( 1+\psi\mu_{i,t} \right),$$

The negative binomial specification was selected because it provided a better fit than the Poisson model, as indicated by lower information criteria (AIC = 53,274.23 for NegBin1 vs. 53,680.42 for Poisson) [12].

The conditional mean $\mu_{i,t}$ was additively decomposed into three components [13]:

$$\mu_{i,t}= \underset{\text{endemic}}{\underbrace{\nu_{i,t}}}+\underset{\text{autorregressive}}{\underbrace{\lambda_{i}Y_{i,t-1}}}+\underset{\text{spatiotemporal}}{\underbrace{\phi_{i}\sum_{j\neq i} w_{ji}Y_{j,t-1}}}$$

where:

- $\nu_{i,t}$ represents the endemic component, capturing persistent structural factors not explained by recent transmission;
- $\lambda_{i}Y_{i,t-1}$ corresponds to the autoregressive (AR) component, reflecting local temporal dependence;
- $\phi_{i}\sum_{j\neq i} w_{ji}Y_{j,t-1}$ denotes the spatiotemporal (neighborhood) component, modeling transmission between municipalities weighted by a spatial connectivity matrix *w_ji_*​.

### **Endemic component and seasonality**

The endemic component was specified with a population offset and harmonic seasonal terms to capture recurrent monthly patterns [13]:

$$\log\left( \nu_{i,t} \right)=\alpha^{\left( \nu\right)}+\beta_{t}+\sum_{k=1}^{2} \left[ \gamma_{k}\sin\left( \frac{2k\pi t}{12} \right)+\delta_{k}\cos\left( \frac{2k\pi t}{12} \right) \right]+\log\left( e_{i,t} \right),$$

where *e_i,t_*​ is the population size of municipality *i*, included as an offset, $\alpha^{\left( \nu\right)}$ is the intercept, and *t* represents time. First- and second-order seasonal harmonics (12- and 6-month cycles) were included using the *addSeason2formula* function, allowing for flexible representation of complex seasonal patterns in monthly data [10].

### **Epidemic components**

The epidemic components were specified with time-constant intercepts, following the standard *hhh4* formulation [10]:

$$\log\left( \lambda_{i} \right)=\alpha^{\left( \lambda\right)} ,\quad\quad\log\left( \phi_{i} \right)=\alpha^{\left( \phi\right)}.$$

### **Spatial connectivity**

For the neighborhood component, a **distance-based power-law weight matrix** was constructed from inter-municipal centroid distances [14]:

$$w_{ji}\propto d_{ji}^{-d}\boldsymbol{,}$$

with exponent d=0.5. The weights were row-normalized so that each row summed to one, allowing *w_ji_*​ to be interpreted as the fraction of cases in municipality *j* distributed across potential recipient municipalities *i* [15].

**Model assessment and interpretation**

Model adequacy was assessed using the **Probability Integral Transform (PIT)**, by comparing the empirical distribution of observed values with the posterior predictive distribution, enabling evaluation of model calibration and the detection of systematic deviations (Figure S3) [11].

For visualization and interpretation, municipalities were selected based on the **95th percentile of the mean component contributions** (endemic, autoregressive, and neighborhood components), highlighting areas with the greatest relative contribution of each component over the study period (Figure S2) [10].

## **Bayesian spatial analysis of the determinants of pulmonary tuberculosis**

To investigate demographic, socioeconomic, operational, and epidemiological factors associated with pulmonary tuberculosis incidence, we fitted hierarchical Bayesian models, initially without a spatial component and subsequently incorporating explicit spatial dependence [16]. All analyses were conducted in R (version 4.4.2. R Foundation for Statistical Computing, Vienna, Austria) using the Integrated Nested Laplace Approximation (INLA) approach, with support from the *INLA* and *INLAOutputs* packages [17].

### **Non-spatial Bayesian models**

We first fitted non-spatial Bayesian models to estimate associations between covariates and municipal pulmonary tuberculosis incidence. Let $Y_{i}$ denote the number of notified cases in municipality *i* and $N_{i}$​ its resident population. We assumed [18]:

$$Y_{i}\sim Poisson\left( \mu_{i} \right),$$

$$log\left( \mu_{i} \right)=log\left( N_{i} \right)+\beta_{0}+\sum_{k=1}^{p} \beta_{k}X_{ik},$$

where $log\left( N_{i} \right)$ was included as an offset and $X_{ik}$​ represents the covariates for municipality *i*. Models were estimated using INLA, with vague normal priors specified for the intercept and fixed effect coefficients $\beta_{k}$​ with mean zero and low precision, reflecting minimal prior information. Estimates were expressed as relative risks defined as exp(*β*) with 95% credible intervals (CrIs) (i.e., 2.5th and 97.5th percentiles) obtained from the posterior distributions [16].

Before multivariable modelling, multicollinearity among explanatory variables was assessed using the Spearman correlation matrix among covariates, as presented in Table S2, to identify strong monotonic associations and potential redundancies [12]. We also examined variance inflation factors (VIF) and tolerance statistics, adopting cut-off values greater than 5 for VIF and less than 0.20 for tolerance (Table S3) [19]. Based on this assessment, the variable number of physicians per 100,000 inhabitants was excluded due to its high correlation with the number of nurses per 100,000 inhabitants. Similarly, average per capita household income and the proportion of the population vulnerable to poverty were excluded from the multivariable analysis due to high collinearity with other socioeconomic indicators. Continuous variables were then standardized using z-scores [(X*_ij_* −mean(X*_j_* ))/SD (X*_j_*)], so that estimated coefficients represented the effect associated with a one standard deviation (SD) increase in incidence [12].

After the univariable stage, we constructed the non-spatial multivariable model using a forward stepwise selection procedure, guided primarily by the Deviance Information Criterion (DIC) and secondarily by the Watanabe Akaike Information Criterion (WAIC) [20]. We assessed residual spatial dependence by applying the Global Moran index to standardized residuals from the non-spatial model [21]. The detection of significant spatial autocorrelation motivated the inclusion of a spatial component in the subsequent modeling stage [18].

### **Spatial Bayesian models**

In the next stage, we fitted spatial Bayesian models explicitly incorporating spatial dependence between municipalities [16]. Although the data covered a long temporal period from 2001 to 2024, we did not perform spatiotemporal modeling with time varying covariates because most explanatory variables were not consistently available throughout the entire historical series. Several socioeconomic covariates were available only for the 2000 and 2010 demographic censuses, while information from the 2022 census was not yet fully accessible. In addition, some variables began to be collected only in specific years and were unavailable for earlier periods. Given the long time series analyzed, artificially repeating constant values over time was considered methodologically inappropriate, as it could introduce bias and compromise inferential validity. Therefore, we adopted an exclusively spatial approach, which was more appropriate for investigating structural associations between determinants and disease incidence [18].

We modeled the number of notified cases in each municipality assuming a Poisson distribution, with the logarithm of the municipal population included as an offset. The spatial model was specified using the BYM2 (Besag–York–Mollié) reparameterization, which decomposes residual spatial heterogeneity into two components: a structured spatial effect capturing autocorrelation among neighboring areas and an unstructured effect representing non-spatial random variability [22,23]. The structured component was defined using a conditional autoregressive specification based on a queen contiguity neighborhood matrix:

$$log\left( \mu_{i} \right)=log\left( N_{i} \right)+\beta_{0}+\sum_{k=1}^{p} \beta_{k}X_{ik}+u_{i} +\upsilon_{i},$$

where $u_{i}$ denotes the structured spatial effect and $\upsilon_{i}$ ​the unstructured effect.

We adopted penalized complexity priors for the BYM2 hyperparameters [22]. The parameter *ϕ*, which controls the proportion of variance attributed to the structured spatial component, was assigned a penalized complexity prior specified such that Pr(*ϕ* > 0.5) = 2/3, favoring parsimonious models with moderate spatial dependence. For the global precision of the spatial effect, we used a penalized complexity prior defined as Pr(*sd*  > 1) = 0.01, penalizing large residual variance values. Fixed effects were assigned vague normal priors after covariate standardization so that estimates represented the effect associated with a one standard deviation increase [24].

We initially fitted univariable spatial models for each candidate covariate. We then constructed the multivariable spatial model using a forward selection procedure, guided primarily by reductions in the DIC and secondarily by the WAIC. The inclusion of additional covariates required a minimum reduction of two units in the DIC to ensure a balance between model fit and parsimony. The procedure was stopped when no additional variable produced a meaningful improvement in the information criteria [20,25].

We evaluated the use of a negative binomial distribution to accommodate potential residual overdispersion. However, this specification did not improve DIC or WAIC values compared with the Poisson model, which was retained as the final specification [18]. We assessed model adequacy by examining the dispersion parameter, Pearson residuals, and the Global Moran index applied to standardized residuals, confirming the absence of residual spatial autocorrelation after inclusion of the spatial component (Table 2 and Figure S4).

# Supplementary results

**Table S2.** Spearman correlation matrix of the social determinants analyzed in the study.

| **ID** | **Variables** | **1** | **2** | **3** | **4** | **5** | **6** | **7** | **8** | **9** | **10** | **11** | **12** | **13** | **14** | **15** | **16** | **17** | **18** | **19** |
| --- | --- | --- | --- | --- | --- | --- | --- | --- | --- | --- | --- | --- | --- | --- | --- | --- | --- | --- | --- | --- |
| 1 | Number of physicians (per 1,000 inhabitants) | 1.00 |  |  |  |  |  |  |  |  |  |  |  |  |  |  |  |  |  |  |
| 2 | Number of nurses (per 1,000 inhabitants) | **0.59** | 1.00 |  |  |  |  |  |  |  |  |  |  |  |  |  |  |  |  |  |
| 3 | GDP per capita (US$) | **0.32** | **0.35** | 1.00 |  |  |  |  |  |  |  |  |  |  |  |  |  |  |  |  |
| 4 | Municipal health expenditure (per capita) | 0.03 | **0.18** | 0.41 | 1.00 |  |  |  |  |  |  |  |  |  |  |  |  |  |  |  |
| 5 | Families benefiting from *Bolsa Família* | **0.40** | **0.23** | 0.02 | **-0.66** | 1.00 |  |  |  |  |  |  |  |  |  |  |  |  |  |  |
| 6 | Adequate sanitation coverage (%) | 0.06 | **0.16** | -0.02 | -0.09 | **0.13** | 1.00 |  |  |  |  |  |  |  |  |  |  |  |  |  |
| 7 | Proportion of individuals aged 65+ | **0.14** | **0.15** | **-0.17** | **0.13** | **-0.20** | 0.09 | 1.00 |  |  |  |  |  |  |  |  |  |  |  |  |
| 8 | Sex ratio | **-0.44** | **-0.32** | -0.11 | 0.09 | **-0.29** | **-0.19** | **-0.38** | 1.00 |  |  |  |  |  |  |  |  |  |  |  |
| 9 | Coverage of primary care (%) | **0.14** | 0.07 | 0.10 | 0.05 | 0.03 | 0.09 | **0.15** | -0.09 | 1.00 |  |  |  |  |  |  |  |  |  |  |
| 10 | Gini index (%) | 0.01 | 0.04 | 0.08 | 0.00 | **0.17** | 0.03 | **-0.37** | **0.18** | -0.08 | 1.00 |  |  |  |  |  |  |  |  |  |
| 11 | % of vulnerable to poverty | **-0.53** | **-0.45** | **-0.36** | -0.07 | -0.23 | -0.11 | **-0.40** | **0.56** | **-0.14** | 0.35 | 1.00 |  |  |  |  |  |  |  |  |
| 12 | Unemployment rate | 0.05 | **0.14** | **0.15** | 0.11 | -0.02 | 0.02 | -0.02 | -0.06 | 0.08 | 0.11 | 0.01 | 1.00 |  |  |  |  |  |  |  |
| 13 | Household crowding | **-0.14** | **-0.19** | -0.09 | -**0.22** | **0.19** | -0.05 | **-0.56** | **0.33** | -0.10 | **0.22** | **0.44** | -0.20 | 1.00 |  |  |  |  |  |  |
| 14 | Municipal Human Development Index | **0.49** | **0.45** | **0.39** | 0.09 | **0.22** | 0.09 | **0.22** | **-0.44** | 0.17 | **-0.18** | **-0.75** | 0.17 | **-0.40** | 1.00 |  |  |  |  |  |
| 15 | Illiteracy rate of those aged ≥18 years | **-0.27** | **-0.40** | **-0.54** | **-0.25** | -0.09 | 0.04 | 0.08 | 0.08 | 0.00 | -0.11 | **0.40** | -0.28 | **0.29** | **-0.65** | 1.00 |  |  |  |  |
| 16 | Social Vulnerability Index | **-0.24** | **-0.21** | **-0.21** | **-0.18** | 0.03 | -0.11 | **-0.29** | **0.26** | -0.05 | **0.22** | **0.56** | 0.14 | **0.44** | **-0.47** | **0.28** | 1.00 |  |  |  |
| 17 | Average household income per capita (US$) | **0.50** | **0.46** | **0.44** | 0.02 | **0.33** | 0.11 | -0.05 | **-0.37** | 0.13 | **0.17** | **-0.58** | 0.34 | **-0.20** | **0.64** | **-0.50** | **-0.25** | 1.00 |  |  |
| 18 | AIDS incidence rate (per 100,000 inhabitants) | **0.39** | **0.25** | 0.07 | -0.05 | **0.18** | **0.15** | **0.21** | **-0.50** | 0.13 | **-0.20** | **-0.40** | 0.00 | **-0.23** | **0.39** | -0.06 | **-0.22** | **0.26** | 1.00 |  |
| 19 | Proportion of Black and Brown population | 0.01 | **0.15** | -0.11 | 0.03 | -0.06 | 0.02 | -0.03 | 0.06 | 0.02 | 0.03 | 0.05 | 0.10 | 0.03 | 0.09 | **-0.15** | **0.19** | 0.09 | -0.03 | 1.00 |

*Abbreviations:* GDP, gross domestic product; AIDS, Acquired Immunodeficiency Syndrome.

*Note:* Values in bold indicate statistically significant correlations (p-value < 0.05).

**Table S3.** Variance inflation factors and tolerance before and after exclusion of collinear variables.

| **Variables** | **Before** | | **After** | |
| --- | --- | --- | --- | --- |
|  | **VIF** | **Tolerance**  **(1/VIF)** | **VIF** | **Tolerance**  **(1/VIF)** |
| Number of physicians (per 1,000 inhabitants) | 4.812 | 0.208 | – | – |
| Number of nurses (per 1,000 inhabitants) | 2.868 | 0.349 | 1.894 | 0.528 |
| GDP per capita (US$) | 1.753 | 0.571 | 1.635 | 0.612 |
| Municipal health expenditure (per capita) | 1.417 | 0.706 | 1.420 | 0.704 |
| Families benefiting from *Bolsa Família* | 2.051 | 0.487 | 1.570 | 0.637 |
| Adequate sanitation coverage (%) | 1.150 | 0.870 | 1.111 | 0.900 |
| Proportion of individuals aged 65+ | 2.655 | 0.377 | 2.356 | 0.425 |
| Sex ratio | 2.217 | 0.451 | 1.972 | 0.507 |
| Coverage of primary care (%) | 1.043 | 0.959 | 1.042 | 0.960 |
| Gini index (%) | 2.316 | 0.432 | 1.292 | 0.774 |
| % of vulnerable to poverty | 6.078 | 0.165 | – | – |
| Unemployment rate | 1.500 | 0.667 | 1.235 | 0.810 |
| Household crowding | 2.192 | 0.456 | 2.148 | 0.465 |
| Municipal Human Development Index | 6.397 | 0.156 | 4.706 | 0.213 |
| Illiteracy rate of those aged ≥18 years | 2.366 | 0.423 | 3.394 | 0.295 |
| Social Vulnerability Index | 1.882 | 0.531 | 1.916 | 0.522 |
| Average household income per capita (US$) | 6.122 | 0.163 | – | – |
| AIDS incidence rate (per 100,000 inhabitants) | 1.678 | 0.596 | 1.666 | 0.600 |
| Proportion of Black and Brown population | 1.159 | 0.863 | 1.147 | 0.872 |

*Note:* Multicollinearity was considered potentially problematic when variance inflation factor (VIF) values exceeded 5, corresponding to tolerance values below 0.20.

*Abbreviations:* GDP, gross domestic product; AIDS, Acquired Immunodeficiency Syndrome. VIF, Variance Inflation Factor.

| Table S4. Epidemiological characteristics of pulmonary tuberculosis cases in the state of Piauí, Brazil, 2001–2024. | |
| --- | --- |
| **Variables** | **PTB cases** |
|  | **N (%)** |
| **Total** | **17,186 (100.0)** |
| **Period of diagnosis** |  |
| 2001-2008 | 7,344 (42.7) |
| 2009-2016 | 4,853 (28.2) |
| 2017-2024 | 4,989 (29.0) |
| **Health region** |  |
| Carnaubais | 879 (5.1) |
| Chapada das Mangabeiras | 571 (3.3) |
| Chapada Vale do Rio Itaim | 486 (2.8) |
| Cocais | 2,030 (11.8) |
| Entre Rios | 7,338 (42.7) |
| Planicie Litoranea | 2,021 (11.8) |
| Serra da Capivara | 554 (3.2) |
| Tabuleiros do Alto Parnaiba | 265 (1.5) |
| Vale do Caninde | 466 (2.7) |
| Vale do Rio Guaribas | 1,227 (7.1) |
| Vale do Sambito | 480 (2.8) |
| Vale dos Rios Piaui e Itaueiras | 863 (5.0) |
| Ignored | 6 (0.0) |
| **Age, years** |  |
| Mean (SD) | 45.2 (18.9) |
| Median (IQR) | 44.0 (30.0-59.0) |
| **Age group (years)** |  |
| 0-9 | 201 (1.2) |
| 10-19 | 1,071 (6.2) |
| 20-29 | 2,921 (17.0) |
| 30-39 | 2,967 (17.3) |
| 40-49 | 3,082 (17.9) |
| 50-59 | 2,684 (15.6) |
| 60-69 | 2,148 (12.5) |
| 70-79 | 1,495 (8.7) |
| ≥80 | 616 (3.6) |
| **Sex** |  |
| Male | 11,210 (65.2) |
| Female | 5,976 (34.8) |
| **Self-reported race/ethnicity** |  |
| White | 2,013 (11.7) |
| Brown or mixed | 10,518 (61.2) |
| Black | 1,913 (11.1) |
| Asian | 271 (1.6) |
| Indigenous | 44 (0.3) |
| Ignored | 2,427 (14.1) |
| **Education (years)** |  |
| Illiterate | 3,502 (20.4) |
| 1-3 | 3,619 (21.1) |
| 4-7 | 3,647 (21.2) |
| 8-11 | 1,995 (11.6) |
| ≥12 | 1,847 (10.7) |
| Ignored | 2,576 (15.0) |
| **HIV status** |  |
| Negative | 6,547 (38.1) |
| Positive | 746 (4.3) |
| Unknown | 9,893 (57.6) |
| **AIDS** |  |
| No | 8,934 (52.0) |
| Yes | 668 (3.9) |
| Ignored | 7,584 (44.1) |
| **Alcohol user** |  |
| No | 9,099 (52.9) |
| Yes | 1,866 (10.9) |
| Ignored | 6,221 (36.2) |
| **Smoking** |  |
| No | 4,537 (26.4) |
| Yes | 1,141 (6.6) |
| Ignored | 11,508 (67.0) |
| **Drug user** |  |
| No | 5,172 (30.1) |
| Yes | 472 (2.7) |
| Ignored | 11,542 (67.2) |
| **Diabetes mellitus** |  |
| No | 9,569 (55.7) |
| Yes | 1,305 (7.6) |
| Ignored | 6,312 (36.7) |
| **Mental disorder** |  |
| No | 10,457 (60.8) |
| Yes | 337 (2.0) |
| Ignored | 6,392 (37.2) |
| **Other comorbidities** |  |
| No | 7,856 (45.7) |
| Yes | 1,829 (10.6) |
| Ignored | 7,501 (43.6) |
| **Bacteriological status** |  |
| Not confirmed | 6,111 (35.6) |
| Confirmed | 11,075 (64.4) |

*Note:* Self-reported race or ethnicity, classified as Branco (White), Preto (Black), Pardo (Brown or mixed), Amarelo (Asian) or Indígena (Indigenous).

*Abbreviations:* SD, standard deviation; IQR, interquartile range.

**Table S5.** Global spatial autocorrelation analysis of pulmonary tuberculosis in the state of Piauí, Brazil, 2001-2024.

| **Study period** | **Moran’s I** | **z-score** | **p-value^*^** | **Distribution pattern** |
| --- | --- | --- | --- | --- |
| 2001-2008 | 0.214 | 5.07 | 0.001 | Cluster |
| 2009-2016 | 0.356 | 7.90 | 0.001 | Cluster |
| 2017-2024 | 0.366 | 8.68 | 0.001 | Cluster |
| 2001-2024 | 0.356 | 8.42 | 0.001 | Cluster |

^*^p < 0.05 indicates spatial dependence.


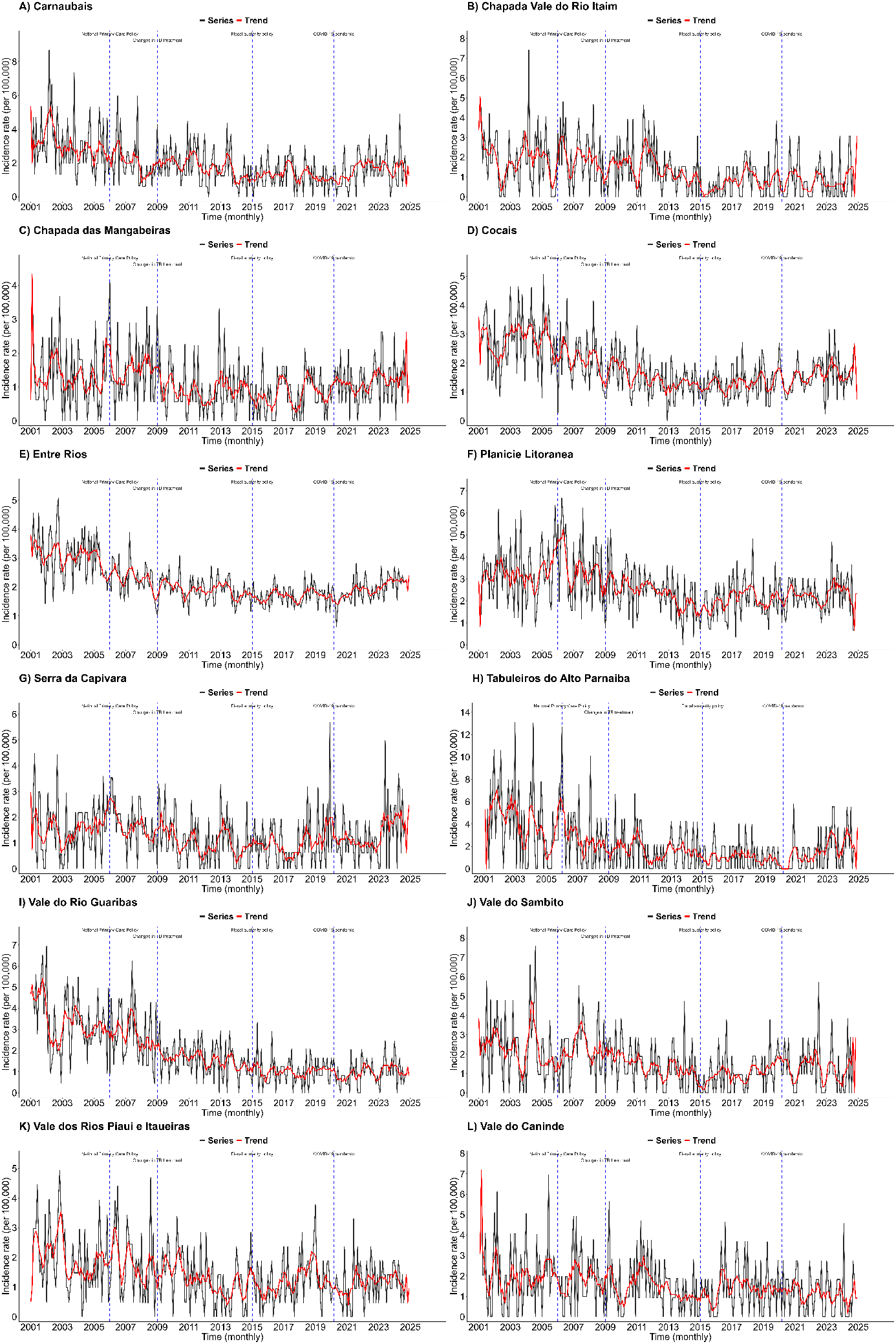
Figure S1. Monthly incidence of pulmonary tuberculosis by health regions in Piauí State, 2001–2024.


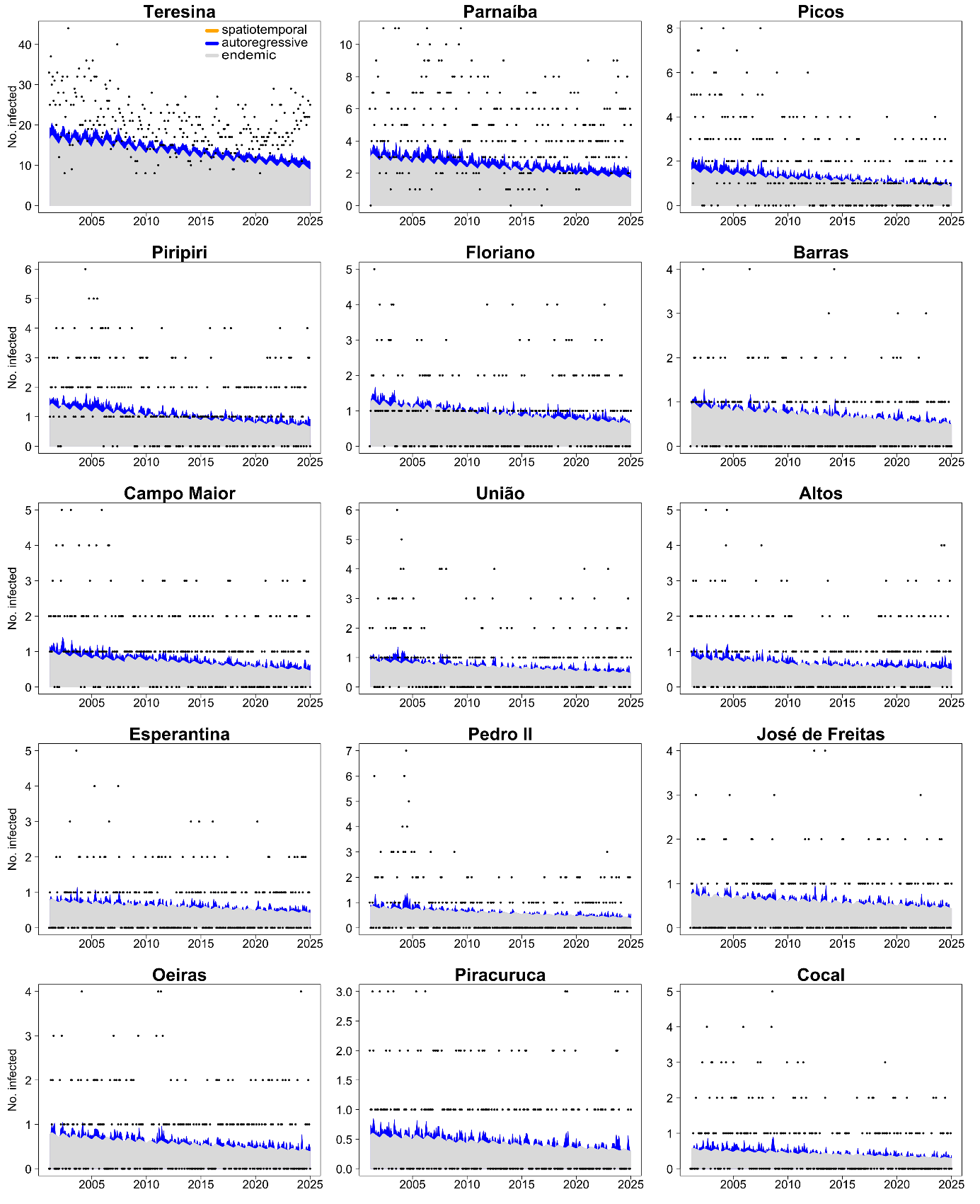


Figure S2. Fitted components in the multivariate time series model for the 15 municipalities with the highest mean contributions in Piauí State, 2001–2024.

*Note:* The black dots represent the monthly counts of incidence, the light grey area shows the endemic component, the blue area shows the autoregressive component, and the yellow area corresponds to the spatiotemporal component.

Table S6. Estimated components of the multivariate time-series model for pulmonary tuberculosis incidence in Piauí, Brazil, 2001–2024.

| **Components** | **Estimate** | **SE** | **95% CI** |
| --- | --- | --- | --- |
| ar.1 | -2.491 | 0.073 | -2.634 to -2.347 |
| ne.1 | -20.770 | 767.500 | -1525.004 to 1483.456 |
| end.1 | -10.690 | 0.020 | -10.724 to -10.647 |
| end.t | -0.0027 | 0.0001 | -0.003 to -0.0025 |
| end.sin(2πt/12) | 0.024 | 0.014 | -0.004 to 0.052 |
| end.cos(2πt/12) | -0.045 | 0.014 | -0.073 to -0.018 |
| end.sin(4πt/12) | -0.007 | 0.014 | -0.034 to 0.021 |
| end.cos(4πt/12) | -0.010 | 0.014 | -0.037 to 0.018 |
| overdispersion | 0.235 | 0.021 | 0.195 to 0.276 |

*Abbreviations:* SE, standard error, CI, confidence interval.

*Note:* ar.1 denotes the first-order autoregressive term, capturing short-term temporal dependence in pulmonary tuberculosis incidence. ne.1 represents the first-order spatial neighbourhood effect, accounting for spatial dependence between adjacent areas. end.1 corresponds to the intercept (baseline level) of the endemic component of the model. end.t indicates the linear temporal trend of the endemic component, reflecting long-term changes in incidence over time. end.sin(2πt/12) and end.cos(2πt/12) are first-order seasonal sine and cosine terms, respectively, modelling annual seasonal variation in incidence. end.sin(4πt/12) and end.cos(4πt/12) are second-order seasonal sine and cosine terms, respectively, capturing semi-annual seasonal patterns.


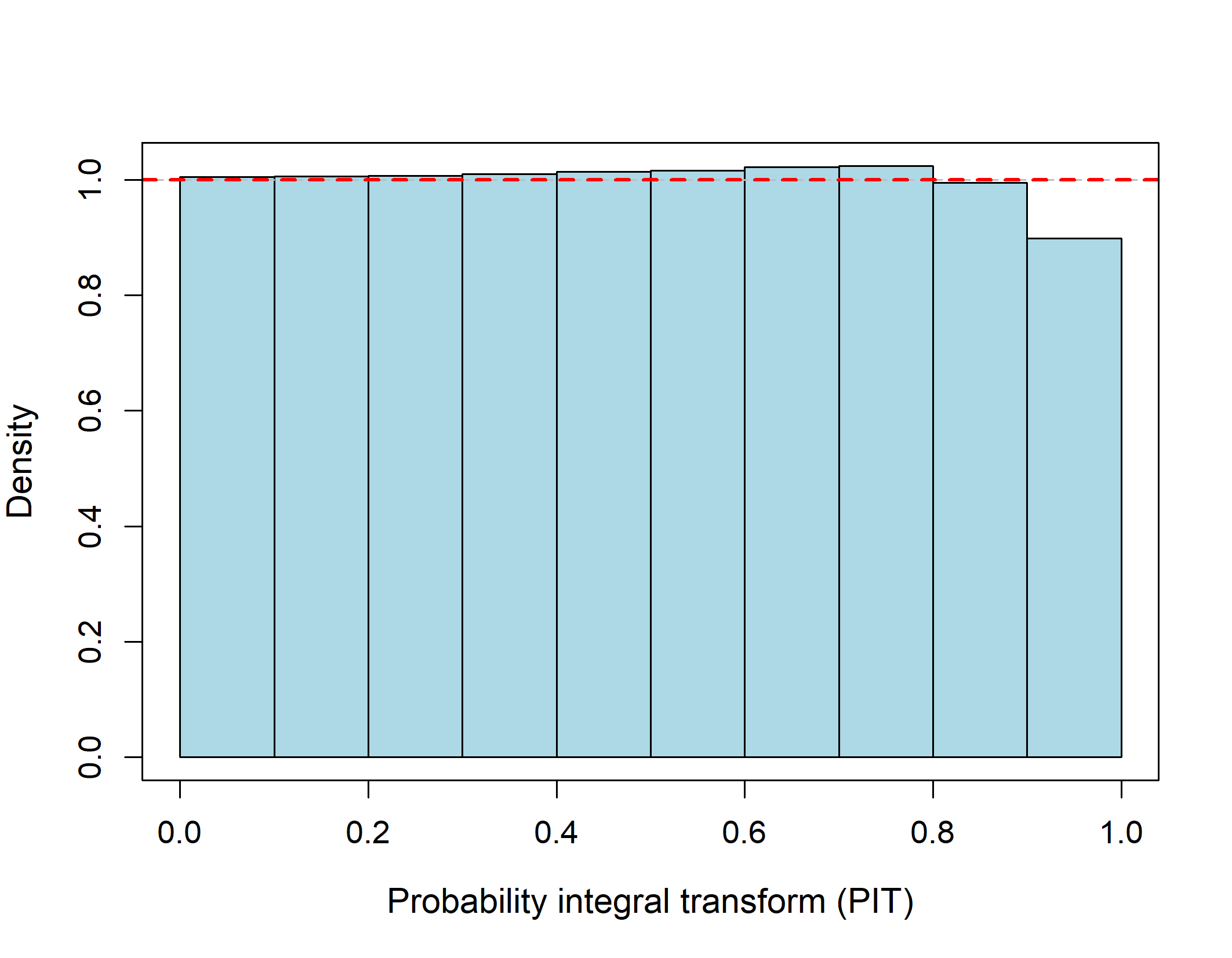


Figure S3. Probability integral transform (PIT) histogram for time-series model calibration.

Table S7. Effect estimates from univariate and multivariable Bayesian non-spatial models of pulmonary tuberculosis determinants in the state of Piauí, Brazil, 2001–2024.

| **Variables** | **Mean (SD)** | **RR** | **95% CrI** | **DIC** | **WAIC** |
| --- | --- | --- | --- | --- | --- |
| **Univariate models for PTB** |  |  |  |  |  |
| Demographic |  |  |  |  |  |
| Sex ratio (M/F) | 101.3 (4.8) | 0.85 | 0.84-0.86 | 2381.44 | 5783.08 |
| Proportion of individuals aged 65+ | 9.3 (11.7) | 0.90 | 0.89-0.92 | 2577.47 | 9900.80 |
| Proportion of Black and Brown population | 0.79 (0.09) | 0.89 | 0.87-0.91 | 2302.84 | 6089.45 |
| Household crowding | 32.3 (6.7) | 0.90 | 0.88-0.91 | 2341.77 | 4348.24 |
| Adequate sanitation coverage (%) | 15.1 (15.8) | 1.11 | 1.10-1.13 | 2348.81 | 7056.17 |
| Socioeconomic |  |  |  |  |  |
| Municipal Human Development Index | 0.57 (0.04) | 1.10 | 1.09-1.11 | 2391.42 | 5580.72 |
| Gini index (%) | 54.0 (0.05) | 1.10 | 1.08-1.12 | 2605.40 | 9892.15 |
| Social Vulnerability Index | 0.47 (0.07) | 0.87 | 0.86-0.88 | 2417.79 | 7113.13 |
| Illiteracy rate of those aged ≥18 year | 32.9 (6.6) | 0.90 | 0.89-0.91 | 2503.97 | 5700.24 |
| Unemployment rate | 7.7 (4.7) | 1.09 | 1.07-1.11 | 2401.70 | 5297.12 |
| % of vulnerable to poverty | 69.5 (8.3) | 0.89 | 0.89-0.90 | 2319.45 | 7026.46 |
| Average household income per capita (US$) | 229.2 (8.8) | 1.09 | 1.08-1.09 | 2357.59 | 7088.31 |
| GDP per capita (US$) | 3,019.1 (3,564.2) | 1.11 | 1.09-1.12 | 2381.41 | 6486.32 |
| Families benefiting from *Bolsa Família* | 2,696.2 (7,233.8) | 1.03 | 1.02-1.03 | 2180.66 | 3485.95 |
| Operational and epidemiological |  |  |  |  |  |
| Number of nurses (per 1,000 inhabitants) | 1.4 (0.64) | 1.13 | 1.12-1.14 | 2318.68 | 8832.87 |
| Number of physicians (per 1,000 inhabitants) | 0.9 (0.6) | 1.07 | 1.07-1.08 | 2269.69 | 7960.78 |
| Coverage of primary care (%) | 99.6 (1.48) | 0.95 | 0.94-0.95 | 2393.31 | 2862.69 |
| AIDS incidence rate (per 100,000 inhabitants) | 5.1 (4.2) | 1.10 | 1.10-1.12 | 2071.91 | 5427.99 |
| Municipal health expenditure (per capita) | 248.4 (71.0) | 1.08 | 1.07-1.09 | 2335.00 | 3718.66 |
| **Multivariable model for PTB** |  |  |  | 1800.23 | 2232.34 |
| Socioeconomic |  |  |  |  |  |
| Unemployment rate | 7.7 (4.7) | 1.03 | 1.01-1.05 |  |  |
| Illiteracy rate of those aged ≥18 year | 32.9 (6.6) | 1.05 | 1.02-1.07 |  |  |
| Operational and epidemiological |  |  |  |  |  |
| Number of nurses (per 1,000 inhabitants) | 1.4 (0.64) | 1.08 | 1.06-1.10 |  |  |
| AIDS incidence rate (per 100,000 inhabitants) | 5.1 (4.2) | 1.09 | 1.07-1.10 |  |  |
| **Model diagnostics** |  |  |  |  |  |
| Overdispersion (ĉ) | 2.67 | 2.36-3.04 | |  |  |
| Global Moran’s I, P-value | 0.26 | 0.001 | |  |  |

*Abbreviations*: CrI: credible interval; DIC, deviance information criteria; PTB, pulmonary tuberculosis; RR: relative risk; SD, standard deviation; WAIC, Watanabe–Akaike information criterion.

*Note:* Effect estimates represent the relative change in the incidence rate associated with a one–standard deviation (1 SD) increase in the exposures of interest. RRs with 95% CrI excluding 1 were considered indicative of a statistically significant.


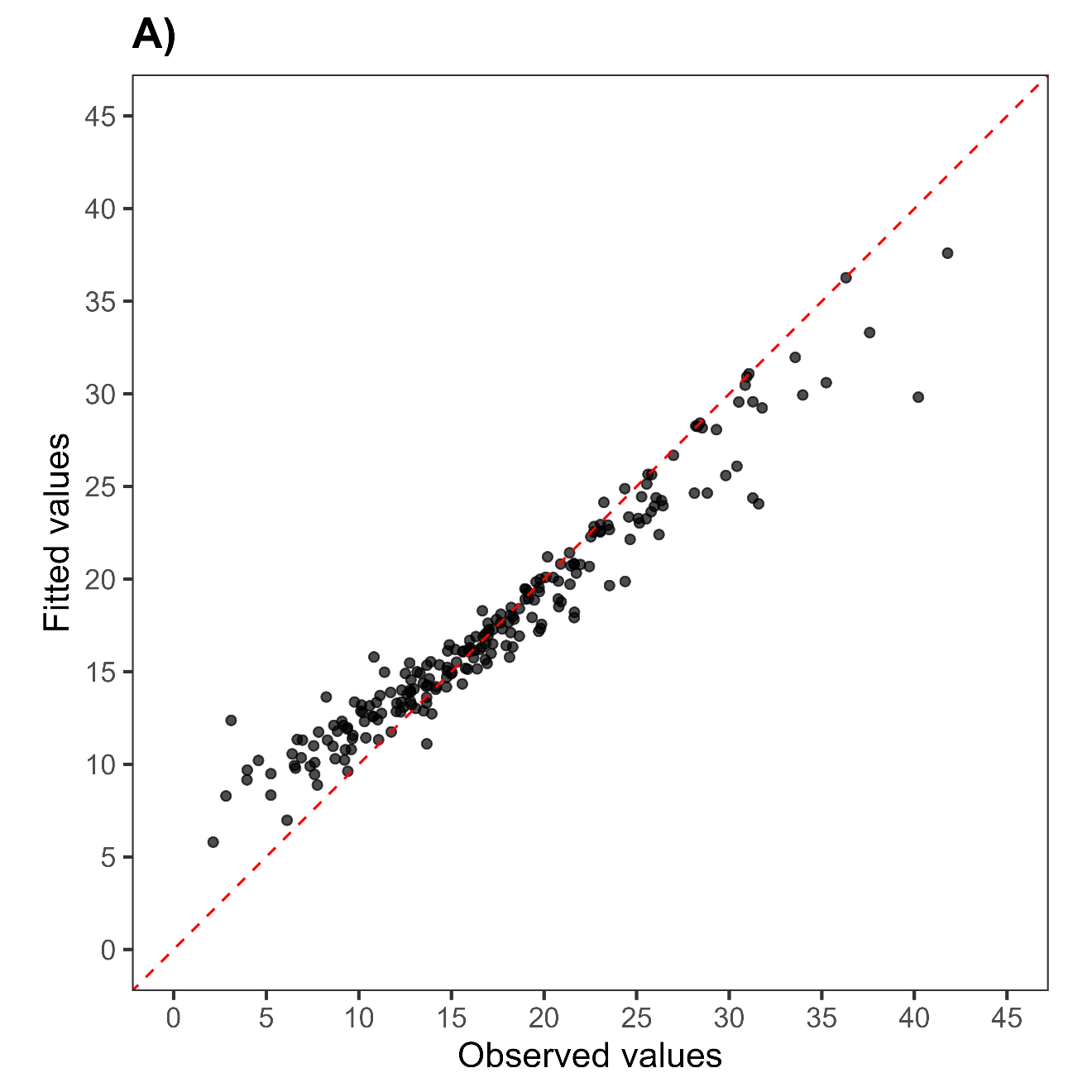


Figure S4. Observed versus fitted values from the Bayesian spatial model for pulmonary tuberculosis incidence in Piauí, Brazil, 2001–2024.

# References

1. Instituto Brasileiro de Geografia e Estatística. Censo demográfico 2022 [Internet]. [cited 2026 June 1]. Available from: https://www.ibge.gov.br/estatisticas/sociais/trabalho/22827-censo-demografico-2022.html

2. Programa das Nações Unidas para o Desenvolvimento. Atlas do desenvolvimento humano no Brasil [Internet]. 2021 [cited 2026 June 1]. Available from: http://www.atlasbrasil.org.br/acervo/biblioteca

3. Instituto Brasileiro de Geografia e Estatística. Censo demográfico 2010 [Internet]. [cited 2026 June 1]. Available from: https://www.ibge.gov.br/estatisticas/sociais/trabalho/22827-censo-demografico-2022.html

4. Instituto Brasileiro de Geografia e Estatística. Produto interno bruto dos municípios: 2022-2023 [Internet]. [cited 2026 Jan 7]. Available from: https://www.ibge.gov.br/estatisticas/economicas/contas-nacionais/9088-produto-interno-bruto-dos-municipios.html

5. Ministério do Desenvolvimento e Assistência Social, Família e Combate à Fome. Bolsa família [Internet]. [cited 2026 Jan 6]. Available from: https://dados.gov.br/dados/conjuntos-dados/bolsa-familia

6. Ministério da Saúde do Brasil. Cadastro nacional de estabelecimentos de saúde - CNES [Internet]. [cited 2026 Jan 6]. Available from: https://cnes.datasus.gov.br/pages/downloads/arquivosBaseDados.jsp

7. Ministério da Saúde do Brasil, Secretaria de Atenção Primária à Saúde. e-Gestor atenção básica - relatórios públicos [Internet]. [cited 2026 Jan 4]. Available from: https://egestorab.saude.gov.br/

8. Ministério da Saúde do Brasil. Informações de saúde (TABNET) – DATASUS [Internet]. [cited 2026 Jan 7]. Available from: https://datasus.saude.gov.br/informacoes-de-saude-tabnet/

9. Ministério da Saúde do Brasil. Sistema de informações sobre orçamentos públicos em saúde - SIOPS [Internet]. [cited 2026 Jan 6]. Available from: https://dados.gov.br/dados/conjuntos-dados/siops

10. Meyer S, Held L, Höhle M. Spatio-temporal analysis of epidemic phenomena using the R package surveillance. J Stat Softw. 2017;77(11):1–55.

11. Held L, Höhle M, Hofmann M. A statistical framework for the analysis of multivariate infectious disease surveillance counts. Stat Model. 2005;5(3):187–99.

12. Tabachnick BG, Fidell LS, Ullman JB. Using multivariate statistics. Seventh edition. New York, NY: Pearson; 2019. 832 p.

13. Held L, Paul M. Modeling seasonality in space‐time infectious disease surveillance data. Biom J. 2012;54(6):824–43.

14. Geilhufe M, Held L, Skrøvseth SO, Simonsen GS, Godtliebsen F. Power law approximations of movement network data for modeling infectious disease spread. Biom J. 2014;56(3):363–82.

15. Meyer S, Held L. Power-law models for infectious disease spread. Ann Appl Stat. 2014;8(3):1612–39.

16. Blangiardo M, Cameletti M. Spatial and spatio‐temporal Bayesian models with R‐INLA. 1st edn. Wiley; 2015.

17. Rue H, Martino S, Chopin N. INLA: Approximate Bayesian inference using Integrated Nested Laplace approximations [Internet]. https://www.r-inla.org/; 2024. Available from: https://www.r-inla.org/

18. Moraga P. Geospatial health data: modeling and visualization with R-INLA and Shiny. Boca Raton, FL: CRC Press; 2020. 274 p. (Chapman & Hall/CRC biostatistics series).

19. O’brien RM. A caution regarding rules of thumb for Variance Inflation Factors. Qual Quant. 2007;41(5):673–90.

20. Gelman A, Hwang J, Vehtari A. Understanding predictive information criteria for Bayesian models. Stat Comput. 2014;24(6):997–1016.

21. Moran PAP. The interpretation of statistical maps. J R Stat Soc Ser B Methodol. 1948;10(2):243–51.

22. Simpson D, Rue H, Riebler A, Martins TG, Sørbye SH. Penalising model component complexity: a principled, practical approach to constructing priors. Stat Sci. 2017;32(1):1–28.

23. Besag J, York J, Mollié A. Bayesian image restoration, with two applications in spatial statistics. Ann Inst Stat Math. 1991;43(1):1–20.

24. Riebler A, Sørbye SH, Simpson D, Rue H. An intuitive Bayesian spatial model for disease mapping that accounts for scaling. Stat Methods Med Res. 2016;25(4):1145–65.

25. Rue H, Martino S, Chopin N. Approximate Bayesian Inference for Latent Gaussian models by using Integrated Nested Laplace Approximations. J R Stat Soc Ser B Stat Methodol. 2009;71(2):319–92.
